# Dense mapping from sparse visual odometry: a lightweight uncertainty-guaranteed depth completion method

**DOI:** 10.3389/frobt.2025.1644230

**Published:** 2025-09-22

**Authors:** Daolong Yang, Xudong Zhang, Haoyuan Liu, Haoyang Wu, Chengcai Wang, Kun Xu, Xilun Ding

**Affiliations:** School of Mechanical Engineering and Automation, Beihang University, Beijing, China

**Keywords:** mapping, deep learning for visual perception, visual odometry, depth completion, uncertainty estimation

## Abstract

**Introduction:**

Visual odometry (VO) has been widely deployed on mobile robots for spatial perception. State-of-the-art VO offers robust localization, the maps it generates are often too sparse for downstream tasks due to insufffcient depth data. While depth completion methods can estimate dense depth from sparse data, the extreme sparsity and highly uneven distribution of depth signals in VO (∼ 0.15% of the pixels in the depth image available) poses signiffcant challenges.

**Methods:**

To address this issue, we propose a lightweight Image-Guided Uncertainty-Aware Depth Completion Network (IU-DC) for completing sparse depth from VO. This network integrates color and spatial information into a normalized convolutional neural network to tackle the sparsity issue and simultaneously outputs dense depth and associated uncertainty. The estimated depth is uncertainty-aware, allowing for the filtering of outliers and ensuring precise spatial perception.

**Results:**

The superior performance of IU-DC compared to SOTA is validated across multiple open-source datasets in terms of depth and uncertainty estimation accuracy. In real-world mapping tasks, by integrating IU-DC with the mapping module, we achieve 50 × more reconstructed volumes and 78% coverage of the ground truth with twice the accuracy compared to SOTA, despite having only 0.6 M parameters (just 3% of the size of the SOTA).

**Discussion:**

Our code will be released at https://github.com/YangDL-BEIHANG/Dense-mapping-from-sparse-visual-odometry/tree/d5a11b4403b5ac2e9e0c3644b14b9711c2748bf9.

## 1 Introduction

Constructing a detailed and accurate map of the environment is a core task in the spatial perception of mobile robots (Malakouti-Khah et al., 2024). Visual odometry (VO) is widely used on mobile robots for perception due to its computational efficiency and adaptability to various environments (Labbé and Michaud, 2022; Aguiar et al., 2022). While state-of-the-art VO provides accurate localization, the resulting sparse depth data often leads to incomplete maps with insufficient spatial information, posing challenges for downstream tasks (Araya-Martinez et al., 2025). With breakthroughs in the computer vision community, sparse depth data can be completed using depth completion approaches (Mathew et al., 2023; Wan et al., 2025), offering a pathway to achieving dense maps in VO. However, the extreme sparsity of depth in VO can only offer limited prior knowledge and still poses significant challenges for depth completion approaches to estimate accurate dense depth for mapping.

Recent developments in depth completion approaches have achieved high accuracy on datasets even with limited input data through carefully designed feature extraction mechanisms and sophisticated network architectures (Chen et al., 2023; Liu et al., 2023; Liu et al., 2022). However, the computational load and memory requirements hinder their practical implementation on mobile robots with limited memory capacity. Additionally, even approaches with high accuracy on datasets still produce a non-negligible number of outliers during inference, leading to false mapping of the environment for robots (Tao et al., 2022). Several previous works have attempted to estimate both dense depth and associated uncertainty within a lightweight network architecture, using the uncertainty to reevaluate depth estimation (Tao et al., 2022; Ma and Karaman, 2018). These works have demonstrated real-world applications in reconstruction, motion planning, and localization. However, most of these works primarily consider inputs from LIDAR or incomplete depth images from depth cameras, which tend to exhibit lower sparsity and a more uniform distribution compared to data obtained from VO.

**FIGURE 1:**
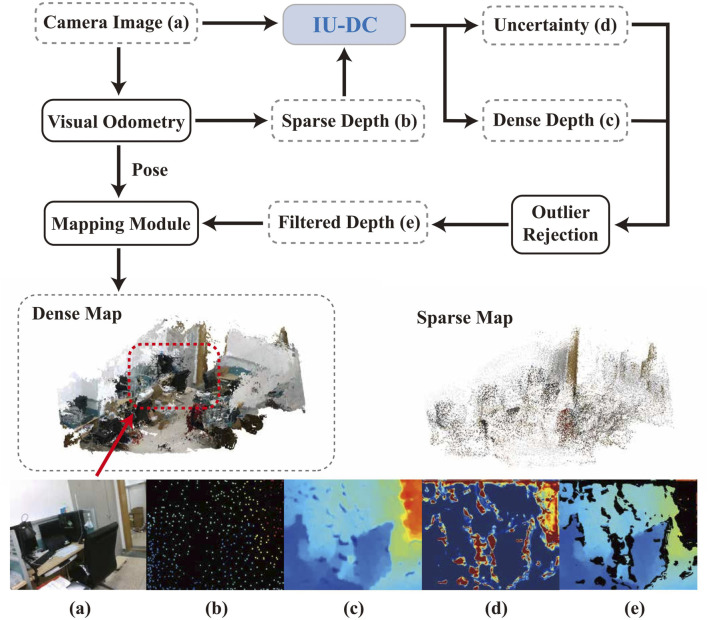
Pipeline of robot mapping with our approach. A dense map of the environment is constructed using only camera images and extremely sparse depth from VO with the proposed IU-DC. **(a)** RGB frame from the camera; **(b)** sparse depth from visual odometry; **(c,d)** dense depth and the associated uncertainty estimated by our network; **(e)** filtered depth obtained using the predicted uncertainty.

Following this method, we propose a novel depth completion network inspired by the normalized convolutional neural network (NCNN) (Eldesokey et al., 2019) to complete the extremely sparse depth data from VO. The pipeline of our method is presented in Figure 1. We name our approach Image-Guided Uncertainty-Aware Depth Completion Network (IU-DC). Our contributions can be summarized as.

- We introduce a Confidence Refine Block that integrates image features into the multi-resolution propagation of NCNN layers, effectively addressing the lack of priors in the sparse input from VO.
- We propose using a map probability density function with the Inverse Sensor Model in the final uncertainty estimation after the last layer of NCNN, enhancing the spatial awareness of the outputs. The accurate uncertainty estimated by IU-DC can then be used to filter out outliers in the depth estimation, providing a more reliable input for mapping.
- The superior performance of IU-DC has been validated against SOTA across multiple datasets in terms of depth and uncertainty estimation. We also conducted mapping experiments on both open-source datasets and our own sequences to support our claims. Our approach reconstructs 50× more volumes than VO, achieving 78% coverage of the ground truth with twice the accuracy compared to SOTA. Despite these improvements, IU-DC occupies only 2.76 MB of memory and can achieve near real-time performance on NVIDIA Xavier NX. We are planning to release the code to support future research.

## 2 Related work

### 2.1 Depth completion with uncertainty awareness

We first briefly review recent developments in depth completion approaches that address both depth and uncertainty estimation. A widely adopted approach involves introducing a second decoder to the original network to output uncertainty. Popović et al. (2021) and Tao et al. (2022) both employed dual decoders to output depth estimation and uncertainty, demonstrating applications in robot mapping and path planning. However, their input sparsity is much lower than that of VO. Qu et al. (2021) introduced a Bayesian Deep Basis Fitting approach that can be concatenated with a base model to generate high-quality uncertainty, even with sparse or no depth input. However, its performance is highly dependent on the base model, making it difficult to achieve in a lightweight network architecture. Additionally, approaches such as ensembling and MC-dropout can estimate uncertainty without modifying the original network (Gustafsson et al., 2020). However, these methods involve a time-consuming inference process, which hinders real-time performance on robots.

Another promising approach is based on the theory of confidence-equipped signals in normalized convolution. Eldesokey et al. (2019) proposed a normalized convolutional neural network (NCNN) that generates continuous confidence maps for depth completion using limited network parameters. They further refined their work to obtain a probabilistic version of NCNN in (Eldesokey et al., 2020). Though the NCNN demonstrates outstanding performance in both depth completion and uncertainty estimation, it can only be used in an unguided manner due to algebraic constraints. This limitation results in performance degradation when the input has high sparsity due to a lack of prior information (Hu et al., 2022). Teixeira et al. (2020) attempted to extend NCNN into an image-guided method by concatenating the image with the outputs from NCNN into another network to generate the final prediction. While this approach improved depth completion accuracy, the resulting uncertainty lacked the continuity inherently modeled by NCNN. In this work, our proposed IU-DC extends NCNN into an image-guided approach to address the sparsity issue while maintaining inherent continuity to generate precise uncertainty estimation.

### 2.2 Depth completion from sparse VO

Several recent works have addressed the challenge of completing sparse depth from VO (Liu et al., 2022) (Wong et al., 2020; Merrill et al., 2021; Wofk et al., 2023). Wong et al. (2020) adopted an unsupervised approach, utilizing a predictive cross-modal criterion to train a network for inferring dense depth. Liu et al. (2022) adopted an adaptive knowledge distillation approach that allows the student model to leverage a blind ensemble of teacher models for depth prediction. Wofk et al. (2023) performed global scale and shift alignment with respect to sparse metric depth, followed by learning-based dense alignment, achieving state-of-the-art performance in depth completion accuracy. Although the sparsity issue of VO has been addressed in depth completion processes, few works are uncertainty-aware and demonstrate evaluations in mapping tasks.

## 3 Methodology

### 3.1 Overall network architecture

**FIGURE 2:**
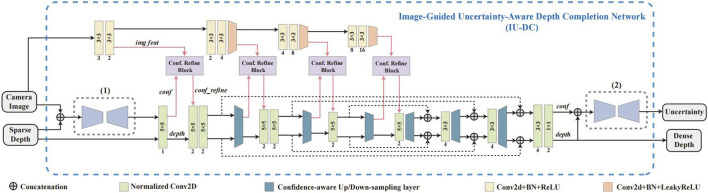
An overview of the proposed IU-DC. **(1)** Input Confidence Estimation Network, **(2)** Model-Based Uncertainty Estimation Network. The middle section with the Confidence Refine Block (Conf. Refine Block) represents the Image-Guided Normalized Convolutional Neural Network.

Our network mainly comprises three main modules: the Input Confidence Estimation Network, which takes camera images and sparse depth as input and estimates the confidence mask input to first NCNN layer; the Image-Guided Normalized Convolutional Neural Network, which uses NCNN as backbone and refines the confidence output from NCNN layers at different resolutions with image features using the proposed Confidence Refine Block; and the Model-based Uncertainty Estimation Network, which takes the estimated depth and confidence output from last NCNN layer to estimates the final output uncertainty for each data. The overall architecture of our network is presented in Figure 2, and the details of each module are explained in the following sections.

### 3.2 Input confidence estimation network

In Eldesokey et al. (2020), the initial confidence mask input into NCNN is learned from the sparse depth using a compact network. However, when the input data becomes sparser and more randomly distributed, confidence estimation may degrade because structure information, such as neighboring objects and sharp edges, is significantly missing (Hu et al., 2022). Sparse depth from VO is always calculated through the KLT sparse optical flow algorithm using corner features (Qin et al., 2018), which have a close correlation with the camera image. To compensate for the missing cues, we utilize both the image and sparse depth together to estimate the input confidence. In the Input Confidence Estimation Network, the image and sparse depth are first concatenated and then input into a compact UNet (Ronnebe et al., 2015) with a Softplus activation at the final layer to generate positive confidence estimations.

### 3.3 Image-guided normalized convolutional neural network

The motivation for adopting NCNN as our backbone lies in its inherent ability to explicitly model confidence propagation. Unlike conventional convolutional networks, NCNN operates on confidence-equipped signals and interpolates missing values in a mathematically principled manner. This capability is particularly valuable under extreme sparsity, such as in VO-derived depth inputs, where the lack of priors makes robust estimation difficult. Moreover, NCNN naturally facilitates uncertainty estimation through confidence propagation, which aligns well with our objective of producing uncertainty-aware depth maps.

Prior studies have shown that image features—especially in regions such as reflective surfaces and occlusion boundaries—often carry rich structural cues that can complement sparse or unreliable depth information (Kendall and Gal, 2017). These features serve as valuable priors for improving confidence estimation, particularly in scenarios where the input depth is extremely sparse and unevenly distributed, as in VO-based depth completion.

However, directly incorporating image features into NCNN is not straightforward. This is because normalized convolution enforces algebraic constraints that require a strict correspondence between the input signal and its associated confidence. Consequently, common practices in image-guided depth completion—such as concatenating image features with the depth signal (Tao et al., 2022; Popović et al., 2021; Eldesokey et al., 2019)—would violate these constraints and compromise the formulation of NCNN.

To leverage this potential without violating NCNN’s constraints, we propose the Confidence Refine Block (CRB). The primary motivation behind CRB is to introduce image guidance indirectly, by refining the intermediate confidence maps produced by NCNN layers. Rather than altering the depth signal directly, CRB enhances the confidence propagation process using gated fusion mechanisms and attention-based refinement. This design preserves the integrity of normalized convolution while effectively injecting contextual priors from the image, leading to improved performance under extreme sparsity.

In this section, we first review the basic concepts of the NCNN, and then introduce the details of how the proposed CRB fits into NCNN.

#### 3.3.1 Normalized convolutional neural network

The fundamental idea of the normalized convolution is to project the confidence-equipped signal $y\in C^{n}$ to a new subspace spanned by a set of basis functions ${\left\{b_{j}\right\}}_{j=0}^{m}$ using the signal with high confidence $c\in R_{+}^{n}$ . Afterwards, the full signal is reconstructed from this subspace, where the less-confident areas are interpolated from their vicinity using a weighting kernel denoted as the applicability function $a\in R_{+}^{n}$ . Thus the image of the signal under the subspace spanned by the basis is obtained as y=Br , where B is a matrix contains all the basis functions and r is a vector of coordinates. These coordinates can be estimated from a weighted least-squares problem (WLS) between the signal y and the basis B (Knutsson and Westin, 1993):

$$\begin{array}{cc} \hat{r} & =\arg\underset{r\in C^{m}}{\min}\|Br-y{\|}_{W}\\ & ={\left(B_{n}^{*}{WB}_{n}\right)}^{-1}B_{n}^{*}Wy,\\ W & =W_{a}\cdot W_{c}=\text{diag}\left(a\right)\cdot \text{diag}\left(c\right). \end{array}$$ (1)

Finally, the WLS solution $\hat{r}$ can be used to estimate the signal:

$$\hat{y}=B\hat{r}.$$

Instead of manually choosing the applicability function, the optimal a in certain scenarios can be learned from NCNN (Eldesokey et al., 2019). This was achieved by using the naïve basis which set $B=1_{n}$ :

$${\hat{r}}_{i}^{l}={\left(1_{n}^{*}W_{a}W_{c}1_{n}\right)}^{-1}1_{n}^{*}W_{a}W_{\mathrm{cy}}=\frac{\langle a^{l}|\left(y^{l-1}⊙c^{l-1}\right)\rangle }{\langle a^{l}|c^{l-1}\rangle },$$ (2)

where $1_{n}$ is a vector of ones, ⊙ is the Hadamard product, ⟨.|.⟩ is the scalar product, ${\hat{r}}_{i}$ is a scalar which is equivalent to the estimated value at the signal ${\hat{y}}_{i}$ . The superscripts l and (l−1) indicate the l−th and (l−1)−th layer of NCNN, respectively. The confidence is propagated as:

$${\hat{c}}_{i}^{l}=\frac{\langle a^{l}|c^{l-1}\rangle }{\langle 1_{n}|a^{l}\rangle },$$ (3)

where the output from one layer is the input to the next layer. If the image feature is directly concatenated with the depth signal y in (Equation 2) to construct a new signal $y^{\prime }$ , its dimensionality increases. Moreover, since c in (Equation 2) is the output of the previous NCNN layer and maintains a strict correspondence with each depth signal y , its dimensions remain consistent. Consequently, the new signal $y^{\prime }$ ‘s dimensions do not match those of c , thereby preventing the application of the Hadamard operation. Another straightforward way to integrate the image feature with the depth signal y is through convolution to form a new signal $y^{\prime }$ . Although this operation resolves the dimensional mismatch, it no longer guarantees the correspondence between $y^{\prime }$ and c . These issues motivate us to design the Confidence Refinement Block to integrate the image feature into NCNN without violating the signal-confidence correspondence.

#### 3.3.2 Confidence refine block

We attempt to utilize the image features to refine, but not entirely alter, the confidence from the NCNN layers, as this would severely violate the correspondence between confidence and signals. Since sparse depth from VO is primarily concentrated in high-texture areas (e.g., object contours) while being sparsely distributed in low-texture regions (e.g., flat walls), this disparity leads to varying contributions of image features to confidence estimation across different areas. Given these challenges, a vanilla convolution (a standard convolution operation with normalization and an activation function) that treats all inputs as valid values is not suitable.

**FIGURE 3:**
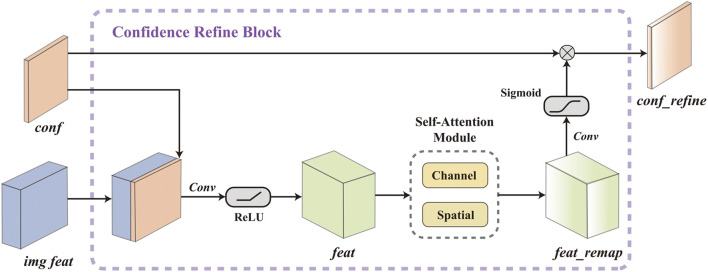
Detailed structure of proposed Confidence Refine Block.

Gated Convolution (Yu et al., 2019), which uses additional convolution kernels to generate gating masks for adaptive feature reweighting, is well-suited to our case. We modified the original form of gated convolution, which originally takes only one feature as input, to simultaneously consider both confidence and image features when calculating the gating signal, as shown in Figure 3. Although sophisticated modality fusion techniques have been proposed in recent years and can be adopted to fuse confidence features with image features (Liu et al., 2023), these methods often rely on complex convolution operations, which increase model complexity and go against our lightweight design. To address this issue, we adopt a straightforward yet effective strategy: first concatenating the two feature maps and encoding them with a lightweight convolution layer, then refining the fused representation using an efficient Self-Attention Module.

Denoting the confidence from NCNN layer as $F_{\mathrm{conf}}$ and image feature as $F_{\mathrm{img}}$ , they have the same size of H×W but with different channel number $C_{\mathrm{conf}}$ and $C_{\mathrm{img}}$ . We first concatenates them into tensor $F_{in}=\left[F_{\mathrm{conf}};F_{\mathrm{img}}\right]$ whose size is $\left(C_{\mathrm{conf}}+C_{\mathrm{img}}\right)\times H\times W$ . Then we use a *Conv* layer, which contains a 2D-convolution layer and a batch normalization layer with a leakyReLU activation layer (to avoid a substantial increase in the number of parameters during feature extraction while maintaining responsiveness to negative values), to encode the concatenated feature tensor:

$$F^{C_{\mathrm{feat}}\times H\times W}=ReLU\left(Conv\left(F_{in}^{\left(C_{\mathrm{conf}}+C_{\mathrm{img}}\right)\times H\times W}\right)\right).$$

however, $C_{\mathrm{conf}}$ and $C_{\mathrm{img}}$ often exhibit large differences, posing a challenge for the encoding process to distinguish between weights from different features. For instance, at the lowest image resolution, $C_{\mathrm{conf}}$ is two while $C_{\mathrm{img}}$ is 16. To address this limitation, we remap the feature map using a self-attention mechanism from (Woo et al., 2018). The feature map $F^{C_{\mathrm{feat}}\times H\times W}$ is first inferred through a 1D channel attention map $M_{c}\in R^{C_{\mathrm{feat}}\times 1\times 1}$ and then through a 2D spatial attention map $M_{s}\in R^{1\times H\times W}$ :

$$\begin{array}{cc} F^{\prime } & =M_{c}\left(F\right)⊗F,\\ F^{\prime \prime } & =M_{s}\left(F^{\prime }\right)⊗F^{\prime }, \end{array}$$

where ⊗ denotes the element-wise multiplication between two tensors. To calculate the final gating signal $G_{c}$ , we decode the remapped feature $F^{\prime \prime }$ using a *Conv* layer followed by a sigmoid activation layer. Finally, the refined confidence $F_{\mathrm{conf}}^{\prime }$ can be obtained by implementing element-wise multiplication.

$$G_{c}=Sigmoid\left(Conv\left(F^{\prime \prime }\right)\right),$$

$$F_{\mathrm{conf}}^{\prime }=F_{\mathrm{conf}}⊗G_{c}.$$

**FIGURE 4:**
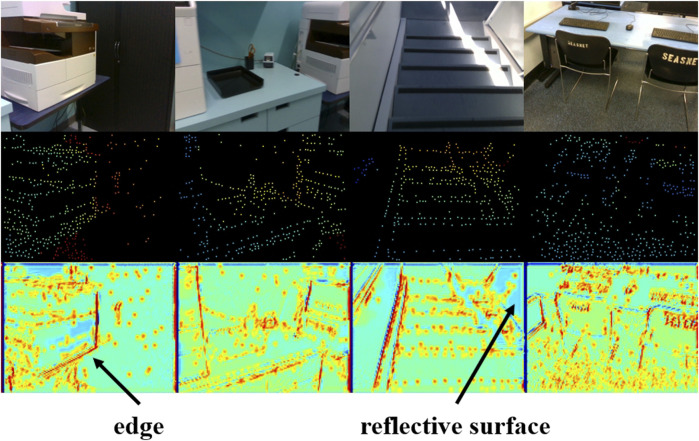
Visualization of the gating signal in the Confidence Refine Block. The first row presents the input image, the second row presents the input sparse depth from VO, and the third shows the corresponding gating signal.

In IU-DC, we integrate one CRB after each confidence-aware down-sampling layer in the NCNN to learn the correlation between confidence and image at different resolutions, as shown in the upper section of Figure 2. We also provide a visualization of the gating signals $G_{c}$ in Figure 4. It can be observed that $G_{c}$ effectively captures semantic features from the image to enhance the confidence map—such as sharp edges and reflective surfaces. Consequently, regions with sparse input signals can be effectively interpolated. This visualization also unveils a hidden relationship between the input depth from VO and the internal propagation within NCNN—depth signals located at more salient object contours tend to have a greater impact on the reconstruction process.

### 3.4 Model-based uncertainty estimation network

In NCNN, the confidence is propagated separately from the depth signal as shown in (Equation 3), which results in a lack of spatial information. For instance, neighbors estimated from larger depth values typically have higher uncertainty compared to those from smaller depth values, which cannot be distinguished by normalized convolution due to the fixed size of the applicability function a .

To address this limitation, we assume that the dense depth output from NCNN forms an occupancy map in the camera frame and follows the probabilistic formulation of the Inverse Sensor Model (ISM) (Agha-Mohammadi et al., 2019). We integrate the confidence output from NCNN as a prior into this ISM-based probability model, thereby enabling the estimation of spatially-aware uncertainty. Furthermore, the entire module can be smoothly trained in an end-to-end manner using the loss function proposed in Section 3.5.

The probability distribution of individual voxel $m^{i}$ can be computed through Bayes’ rule in recursive manner as:

$$\begin{array}{cc} s_{k}^{m^{i}} & =p\left(m^{i}|z_{0:k},x_{0:k}\right)\\ & =\frac{p\left(z_{k}|m^{i},z_{0:k-1},x_{0:k}\right)p\left(m^{i}|z_{0:k-1},x_{0:k}\right)}{p\left(z_{k}|z_{0:k-1},x_{0:k}\right)}, \end{array}$$ (4)

where z is the measurement depth, x is the robot location, k is the steps of iteration. By integrating the ISM formulation,

$$\begin{array}{ll} p\left(z_{k}|m^{i},z_{0:k-1},x_{0:k}\right) & \approx p\left(z_{k}|m^{i},x_{k}\right)\\ & =\frac{p\left(m^{i}|z_{k},x_{k}\right)\;p\left(z_{k}|x_{k}\right)}{p\left(m^{i}|x_{k}\right)}, \end{array}$$

which indicates the occupancy probability given a single measurement, into (Equation 4), and assuming that the robot’s previous trajectory $x_{0:k}$ does not affect the map, we obtain:

$$s_{k}^{m^{i}}=\frac{p\left(m^{i}|z_{k},x_{k}\right)p\left(z_{k}|x_{k}\right)p\left(m^{i}|z_{0:k-1},x_{0:k-1}\right)}{p\left(m^{i}\right)p\left(z_{k}|z_{0:k-1},x_{0:k}\right)}.$$ (5)

Assuming a binary occupancy model for voxels (i.e., each voxel is either occupied $m_{i}=1$ or free $m_{i}=0$ ) and considering only the occupancy map in the camera frame - where the occupancy probability is independent of the robot’s motion, (Equation 5) can be simplified as:

$$s_{k}^{m^{i}}=\frac{p\left(m^{i}=1|z_{k}\right)p\left(m^{i}=0\right)}{p\left(m^{i}=0|z_{k}\right)p\left(m^{i}=1\right)}=\frac{{\text{P}}_{\text{ISM}}^{m^{i}}\left(z_{k}\right)}{{\text{P}}_{\mathrm{prior}}^{m^{i}}},$$ (6)

where ${\text{P}}_{\text{ISM}}^{m^{i}}\left(z_{k}\right)$ indicates the probability of the voxel is occupied given the ISM model with measurement, ${\text{P}}_{\mathrm{prior}}^{m^{i}}$ indicates the prior knowledge.

${\text{P}}_{\text{ISM}}^{m^{i}}\left(z_{k}\right)$ can be approximated by (Loop et al., 2016):

$${\text{P}}_{\text{ISM}}^{m^{i}}\left(z_{k}\right)=\text{H}\left(k_{\sigma }z_{k}^{m^{i}}\right),$$ (7)

where $\text{H}\left(\cdot \right)$ is a cubic curve function maps the measurement into occupancy probability and $k_{\sigma }$ is a scalar. We initialize the $k_{\sigma }$ using a clipping operator to constrain the estimated depth $z_{k}$ within an ideal range [in our case, between 0.1 and 8 in VOID (Wong et al., 2020)]. Next, we refine the scalar by leveraging the spatial dependencies of neighboring depth values, and finally, we map each input $z_{k}^{m^{i}}$ into an occupancy probability by uniformly applying the function $\text{H}\left(\cdot \right)$ across all inputs.

Typically, a fixed ${\text{P}}_{\mathrm{prior}}^{m^{i}}$ for all voxels is assumed during deployment. However, due to various environmental factors, this assumption may not hold. We address this issue by adopting the confidence estimated from NCNN, which encodes both geometric and semantic features, as a strong prior. We construct the ${\text{P}}_{\mathrm{prior}}^{m^{i}}$ for each voxel to represent the heteroscedastic uncertainty in the estimation by formulating WLS problem in (Equation 1) as a special case of the Generalized least-squares (GLS), which offers more flexibility in handling individual variances for each observation (Eldesokey et al., 2020):

$${\hat{r}}_{\text{GLS}}={\left(B^{*}V^{-1}B\right)}^{-1}B^{*}V^{-1}y,$$

where $V={\left(W_{a}W_{c}\right)}^{-1}$ to ensure consistency with the solution in (Equation 2). Then, we utilize the GLS solution ${\hat{r}}_{\text{GLS}}$ to estimate the signal $\hat{y}$ , and the uncertainty of $\hat{y}$ can be obtained as:

$$\begin{array}{cc} \mathrm{cov}\left(\hat{y}\right) & =\mathrm{cov}\left(1_{n}{\hat{r}}_{\text{GLS}}\right)=1_{n}\mathrm{cov}\left({\hat{r}}_{\text{GLS}}\right)1_{n}^{*}\\ & =\sigma^{2}1_{n}{\left(1_{n}^{*}V^{-1}1_{n}\right)}^{-1}1_{n}^{*}\\ & =1_{n}\frac{\sigma^{2}}{\left\langle a|c\right\rangle }1_{n}^{*}, \end{array}$$ (8)

where σ is global variance for each signal. (Equation 8) indicates equal uncertainty for the entire neighborhood under a naïve basis. Since each voxel grid corresponds to the signal center ${\hat{y}}_{i}$ , ${\text{P}}_{\mathrm{prior}}^{m^{i}}$ can be represented as:

$$P_{\mathrm{prior}}^{m^{i}}=\mathrm{cov}\left({\hat{y}}_{i}^{\mathrm{last}}\right)=\frac{\sigma_{i}^{2}}{\left\langle a^{\mathrm{last}}|c^{\mathrm{last}}\right\rangle },$$ (9)

where $\sigma_{i}$ is stochastic noise variance $a^{\mathrm{last}}$ and $c^{\mathrm{last}}$ represent the applicability function and confidence from the last NCNN layer respectively, as discussed in Section 3.3. The noise variance $\sigma_{i}$ can be estimated from the confidence output of the last NCNN layer. By integrating (Equation 7) and (Equation 9) into (Equation 6) and extending it to all the pixels in the depth image, we can estimate the uncertainty using a mapping function $\Phi \left(\cdot \right)$ as follows:

$$s_{k}=\Phi \left(k_{\sigma }z_{k},{\hat{c}}^{\mathrm{last}}\right).$$ (10)

**FIGURE 5:**
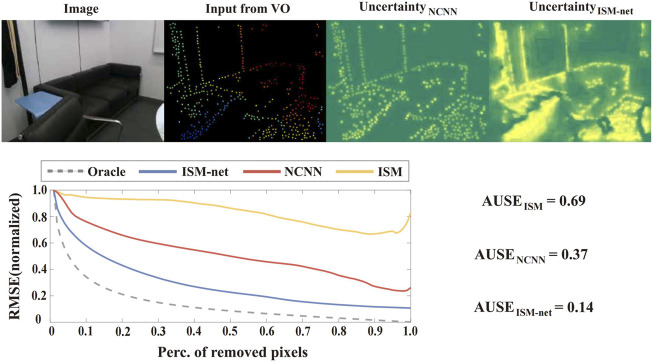
Qualitative and quantitative evaluation of the effectiveness of the Model-based Uncertainty Estimation Network. The upper part of the figure presents the input image, sparse depth from VO, uncertainty estimation from the last NCNN layer, and the final uncertainty estimation from our network (ISM-net). The lower part of the figure illustrates the area under the sparsification error plots (Ilg et al., 2018), where curves closer to the *oracle* represent estimated uncertainty that more closely approximates the real error distribution. ISM-net significantly enhances the uncertainty estimation from NCNN and outperforms the ISM by a large margin.

Our objective is to learn the mapping function $\Phi \left(\cdot \right)$ and the scalar $k_{\sigma }$ in (Equation 10) by concatenating the depth estimation and confidence output from the last NCNN layer into a compact UNet (Ronnebe et al., 2015), as shown in the right section of Figure 2. A direct comparison of the output uncertainty from the Model-based Uncertainty Estimation Network (ISM-net), the NCNN layer (NCNN), and the conventional ISM in (Agha-Mohammadi et al., 2019) (ISM) is presented in Figure 5.

### 3.5 Loss function and training strategy

To achieve the different functions of each module, we require a loss function that enables training the proposed network with uncertainty awareness. Following (Eldesokey et al., 2020) we assume a univariate distribution of each estimated signal under naïve basis ${\hat{y}}_{i}∼N\left({\hat{r}}_{i},s_{i}\right)$ , where ${\hat{r}}_{i}$ is the depth estimation and $s_{i}$ is the uncertainty estimation from IU-DC. The least squares solution in (Equation 2) can be formulated as a maximum likelihood problem of a Gaussian error model. Then the objective is defined as minimizing the negative log likelihood:

$$L\left(w\right)=\frac{1}{N}\overset{N}{\underset{i=1}{\sum }}\frac{\|y_{i}-{\hat{r}}_{i}{\|}^{2}}{s_{i}}+\log\left(s_{i}\right),$$

where w denotes the network parameters.

During the training of our network, we find that initializing the network parameters randomly and training with the loss function L(w) does not guarantee stable convergence. We assume that in the initial training stages, excessively large uncertainty estimations dominate the loss, causing the depth estimation to overcompensate. To address this issue, we adopt a multi-stage training strategy. Initially, we train the network with L2 loss until the network parameters stabilize. Subsequently, we fine-tune the uncertainty output using L(w) .

## 4 Evaluation on NYU and KITTI datasets

We use the standard error metrics of the KITTI depth completion challenge (Uhrig et al., 2017): the Root Mean Square Error (RMSE *m*), the Mean Absolute Error (MAE *m*), the Root Mean Squared Error of the Inverse depth (iRMSE *1/km*), Mean Absolute Error of the Inverse depth (iMAE *1/km*), and the area under sparsification error plots (AUSE) (Ilg et al., 2018) as measure for the accuracy of the uncertainty.

### 4.1 Datasets and setup

*Outdoor*: KITTI dataset (Uhrig et al., 2017) is a large outdoor autonomous driving dataset. We use KITTI depth completion dataset for evaluation, where the training set contains 86k frames, validation set contains 7k frames, and the test set contains 1k frames. The original input depth images have 5% of the pixels available. To simulate the input sparsity of VO, we randomly sample 1k pixels from the raw input depth image, representing approximately 0.2% of the pixels available.

*Indoor*: NYU dataset (Silberman et al., 2012) is an RGB-D dataset for indoor scenes, captured with a Micrasoft Kinect. We use the official split with roughly 48k RGB-D pairs for training and 654 pairs for testing. We randomly sample 500 pixels and 200 pixels from the ground truth depth image, representing available pixels of 0.7% and 0.2%, respectively.

*Setup*: We implement all the networks in PyTorch and train them using the Adam optimizer with an initial learning rate of 0.001 that is decayed with a factor of $10^{-1}$ every 6 epochs follow the training strategy outlined in Section 3.5. All datasets are preprocessed using the same cropping and data augmentation procedures as (Eldesokey et al., 2020).

### 4.2 Comparison to the SOTA

*Baselines:* We propose to obtain dense depth and uncertainty simultaneously, while also considering a lightweight network architecture with low memory consumption suitable for deployment on mobile robots. As baselines, we selected three state-of-the-art networks that meet the requirements: (i) NCONV-AERIAL (Teixeira et al., 2020) is an image guided approach that incorporates NCNN and fuses its output with images to estimate the final depth and uncertainty. (ii) S2D (Tao et al., 2022) is an image-guided approach that concatenates the image and sparse depth image in one encoder and outputs dense depth image and uncertainty from two separate decoders. (iii) PNCNN (Eldesokey et al., 2020) is an unguided approach that also utilizes NCNN as its backbone and has a network structure similar to our approach. We train PNCNN using their open-source code. For S2D, we follow their implementation as described in their paper since they did not release their code. As for NCONV-AERIAL, we use the best results reported in their paper and calculate AUSE using their open-source model.

We initially test all the methods on the KITTI test set with raw input sparsity and the NYU test set with 500 samples as input. We report the accuracy of depth and uncertainty estimation, as well as the number of network parameters in Table 1. S2D and NCONV-AERIAL demonstrate superior accuracy in depth completion compared to PNCNN on the KITTI, attributed to their integration of image features. However, on the NYU dataset where the input sparsity increases to 0.7% , the integration of image features fails to enhance the depth completion performance, even underperforming compared to the unguided approach. Furthermore, both S2D and NCONV-AERIAL exhibit significantly higher AUSE, indicating that the uncertainty output from the networks is not tightly correlated with the actual error distribution. Our proposed IU-DC outperforms PNCNN in depth estimation accuracy and maintains accurate uncertainty estimation across both datasets. This indicates that our modifications enhances overall performance without compromising the uncertainty consistency.

**TABLE 1:** Depth completion results on NYU and KITTI datasets.

Algorithm	RMSE	MAE	iRMSE	iMAE	AUSE ↓
KITTI test set					
*NCONV-AERIAL*	1.01	0.26	—	—	0.39
*S2D*	1.14	0.40	3.97	1.92	0.13
*PNCNN*	1.23	0.28	4.46	1.07	**0.05**
*IU-DC*	**0.94**	**0.23**	**2.72**	**0.97**	0.06
KITTI-1000 samples					
*PNCNN*	2.41	0.70	70.08	2.56	**0.05**
*IU-DC*	**1.59**	**0.50**	**4.87**	**2.02**	**0.05**
NYU-500 samples					
*NCONV-AERIAL*	0.22	0.11	—	—	0.24
*S2D*	0.22	0.16	24.29	16.90	0.30
*PNCNN*	0.18	0.07	24.38	8.77	**0.06**
*IU-DC*	**0.11**	**0.04**	**14.28**	**5.13**	**0.06**
NYU-200 samples					
*PNCNN*	0.24	0.10	56.78	13.82	0.09
*IU-DC*	**0.16**	**0.06**	**20.71**	**8.36**	**0.09**

The number of parameters (#P) for each model is as follows: NCONV-AERIAL: 980 K; S2D: 12 M; PNCNN: 668 K; IU-DC: 689 K. Bold numbers indicate the best performance.

**FIGURE 6:**
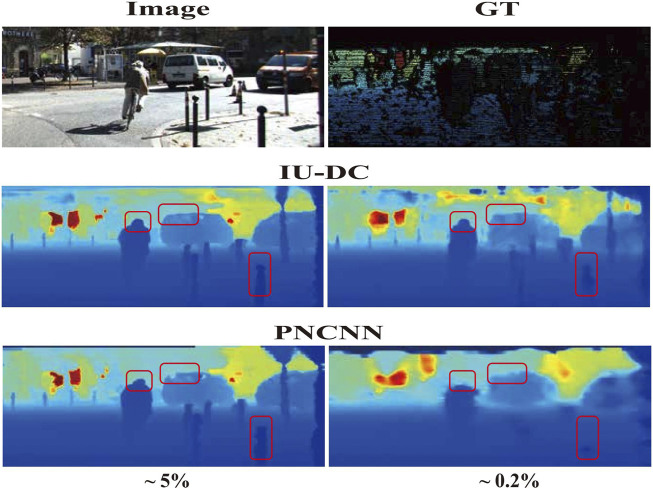
Visualization of depth completion results on the KITTI dataset, where 5% and 0.2% denote the percentage of available pixels in the depth image. PNCNN performs well under low sparsity but exhibits blurriness on the contours of objects and fails to capture most information under high sparsity. In contrast, IU-DC shows more robust performance in both cases.

To simulate the input data from VO with a sparsity of approximately 0.2%, we further test IU-DC and PNCNN on the KITTI dataset with 1,000 samples and the NYU dataset with 200 samples. The iRMSE of PNCNN significantly degraded, being up to 15 times greater than IU-DC in KITTI, indicating the presence of a large number of outliers. This result suggests that when the sparsity becomes extremely high, the neighborhood of the signal cannot be correctly estimated due to the limited receptive field when depth data is the only input source. In contrast, IU-DC achieves robust performance even with this extreme sparsity of input by enriching information around the signal through the integration of image features. This makes IU-DC more suitable for deployment in VO scenarios. To qualitatively observe the results, we present the depth maps estimated from PNCNN and IU-DC on the KITTI dataset in Figure 6. IU-DC captures clearer edges and more detailed contours even when the input sparsity increases significantly.

## 5 Dense mapping from sparse visual odometry

While Section 4 presents evaluations on standard benchmark datasets using synthetically downsampled sparse inputs, in this section we further evaluate IU-DC in real-world visual odometry (VO) scenarios, where the input sparsity and distribution better reflect robot deployment conditions. We additionally assess the impact of uncertainty-aware depth completion on mapping performance.

### 5.1 Evaluation with VO input

#### 5.1.1 Dataset

VOID (Wong et al., 2020) provides real-world data collected using an Intel RealSense D435i camera and the VIO frontend (Fei et al., 2019), where metric pose and structure estimation are performed in a gravity-aligned and scaled reference frame using an inertial measurement unit (IMU). The dataset is more realistic in that no sensor measures depth at random locations. VOID contains 47K training and 800 test samples, with varying levels of input depth density. We adopt 500 points, corresponding to 0.15% of the pixels in the depth image, and follow the published train-test split for evaluation. It is worth noting that our method can generalize to different forms of VO or VIO, as long as the front-end provides metric-scale sparse depth.

#### 5.1.2 Comparison to the SOTA

As baselines, we select two methods that are designed to complete sparse depth from VO, similar to ours but without uncertainty estimation: (i) VOICED (Wong et al., 2020) is an unsupervised method that is among the first to tackle input from VO. (ii) VI-Depth (Wofk et al., 2023) integrates monocular depth estimation with VO to produce dense depth estimates with metric scale. Note that the open-source VI-Depth model was trained with a resolution of 265×265 . We also report the depth completion results at the raw resolution 480×640 in the VOID. The depth completion results are summarized in Table 2. The depth estimated by IU-DC demonstrates higher accuracy compared to VOICED and VI-Depth (480×640) . However, IU-DC underperforms relative to VI-Depth (265×265) , which is the resolution used for training the external monocular depth estimation network in VI-Depth. To demonstrate the effectiveness of accurate uncertainty estimation from the network, we further filter the top 20% of the most uncertain depth values in the depth image and evaluate its accuracy. This is denoted as IU-DC(filtered) in Table 2. After applying the uncertainty-aware filtering, the depth accuracy improves significantly and surpasses other SOTAs by a large margin, e.g., VOICED by 48% and VI-Depth (265×265) by 32% .

**TABLE 2:** Depth completion results on VOID dataset.

Method	MAE	RMSE	iMAE	iRMSE
*VOICED*	124.11	217.43	66.95	121.23
*VI-Depth* (265×265)	94.81	164.36	43.19	**69.25**
*VI-Depth* (480×640)	129.95	210.39	92.23	61.68
*IU-DC (raw)*	102.04	198.29	54.66	103.01
*IU-DC (filtered)*	**62.61**	**111.32**	**37.65**	69.86

^a^ 265×265 and 480×640 denote the input resolutions. Bold numbers indicate the best performance.

#### 5.1.3 Runtime analysis and memory consumption

Runtime and memory consumption are both crucial for deployment on mobile robots to achieve real-time performance. IU-DC exhibits significantly lower parameter counts (0.6M) compared to VOICED (6.4M) and VI-Depth (21M) and only occupies 2.76 MB of memory. We further tested the runtime on NVIDIA GeForce RTX 3050 and NVIDIA Xavier NX with an input resolution of 480×640 . IU-DC runs at 17.5 FPS on the NVIDIA GeForce RTX 3050 and 5.5 FPS on the NVIDIA Xavier NX, while VI-Depth runs at 9 FPS and 3.5 FPS, respectively. IU-DC is nearly twice as fast as VI-Depth. We also tested IU-DC with a lower resolution of 384×384 , achieving 10 FPS on the NVIDIA Xavier NX, which guarantees the update rate for most keyframes in VO. The runtime of IU-DC can be further reduced with engineering enhancements and more advanced computational hardware, e.g., Jetson AGX Orin.

### 5.2 Ablation study

#### 5.2.1 Effect of confidence refine

We first analyze the effect of our proposed Confidence Refinement Block (CRB) by introducing three baselines: (i) integrating image features using vanilla convolution instead of gated convolution (*-w/o gated convolution*); (ii) employing gated convolution without the self-attention module (*-w/o self-attention*); and (iii) using image features to refine the depth signal instead of confidence (*-w/depth refine*). The results are presented in Table 3. Our full model outperforms all baselines across all evaluation metrics, validating that gated convolution extracts more reliable features than vanilla convolution, thereby leading to improved accuracy in depth completion. Furthermore, integrating the self-attention module further enhances performance. We also find that removing the self-attention module in CRB significantly deteriorates the accuracy of uncertainty estimation, increasing the AUSE from 0.14 to 0.49. Moreover, refining confidence yields better results than the depth signal, highlighting the strong correlation between confidence in NCNN layers and images, which supports our motivation for designing CRB.

**TABLE 3:** Ablation study on VOID dataset.

Model	MAE	RMSE	iMAE	iRMSE
Full	**102.04**	**198.29**	**54.66**	**103.01**
Confidence Refine				
*- w/o gated convolution*	117.17	200.18	64.62	112.18
*- w/o self-attention*	104.89	203.85	55.41	107.67
*- w/depth refine*	119.12	210.15	66.72	115.43
ISM Network				
*- w/o ISM model*	144.03	244.00	73.14	123.60
w/VO confidence init	196.44	448.85	921.34	2696.48

Bold numbers indicate the best performance.

**FIGURE 7:**
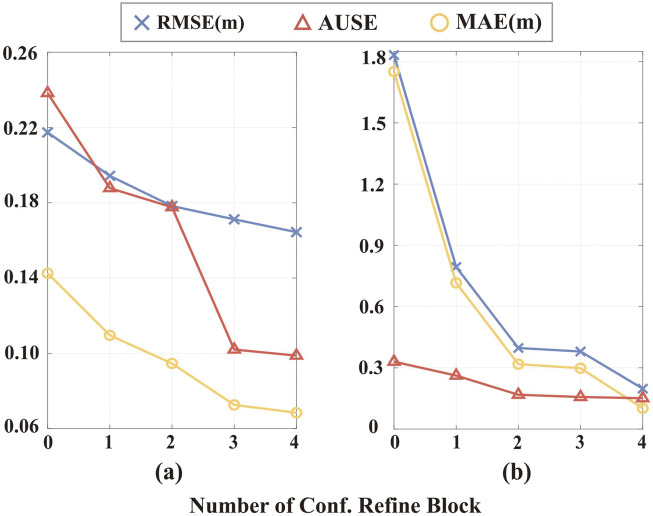
Accuracy of multi-resolution integration of Confidence Refinement Blocks (one per resolution). **(a)** Results on the NYU dataset with 200 samples. **(b)** Results on the VOID dataset.

We further assess whether the multi-resolution integration of CRBs benefits the depth completion process and uncertainty estimation. The results are shown in Figure 7. The major difference between VOID and NYU lies in the input signal distribution. In NYU, the inputs are randomly sampled from the ground truth, whereas in VOID, the inputs are generated from the VO frontend. When integrating more CRBs during inference, we observe an improvement in both depth and uncertainty estimation accuracy. This indicates that CRBs enhance the depth completion process, with their effectiveness becoming more pronounced through multi-resolution integration. It’s worth noting that when no CRBs are integrated into the network, the RMSE and MAE increase by 132% and 208% in NYU, but by 924% and 1716% in VOID. We attribute the substantial deterioration in VOID to the uneven distribution of sparse depth from VO, which validates the crucial role of CRBs in VO depth completion tasks.

#### 5.2.2 Effect of ISM model in uncertainty estimation

**FIGURE 8:**
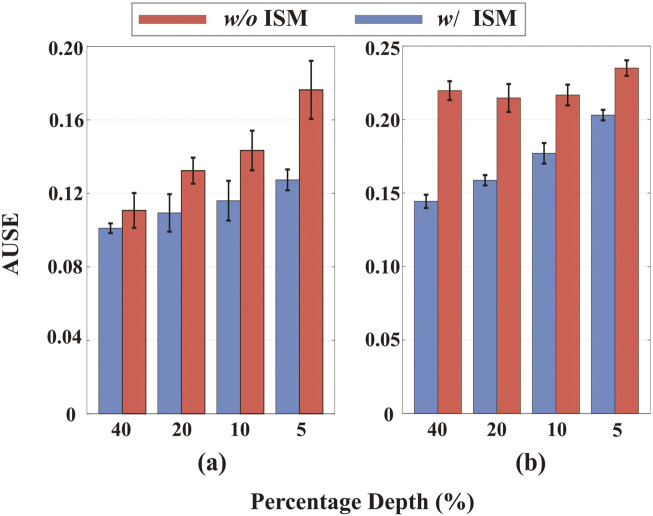
Accuracy of uncertainty estimation with and without the ISM model across different depth value ranges. **(a)** Results on the NYU dataset with 200 samples. **(b)** Results on the VOID dataset. The horizontal axis represents the percentage of top depth values in the depth image, e.g., 40 represents the top 40% of values in the depth image.

IU-DC follows the same uncertainty propagation method as NCNN during the depth completion process but is distinct in its output uncertainty by integrating a map probability density function with the ISM. We validate the role of the ISM model by training a network that only utilize the confidence from the last NCNN layer for uncertainty estimation, the same method as in PNCNN. We evaluate the accuracy of uncertainty for different ranges of depth values and report the error bars in Figure 8. By incorporating the ISM model, the uncertainty estimation improves across different ranges of depth signals, aligning with our motivation discussed in Section 3.4 and confirming that our approach yields more spatially accurate uncertainty outputs. This improvement is consistent whether the input comes from random sampling or VO, validating that the ISM model is robust to the type of input signal and generalizes well across different environments.

Additionally, we report the depth completion accuracy in Table 3 (denoted as *-w/o ISM model*). The results validate that integrating the ISM model into the uncertainty estimation network not only improves uncertainty estimation but also benefits the network training, enabling it to converge to a more accurate depth estimation model.

#### 5.2.3 Does VO uncertainty aid in depth completion?

We are also interested in whether the uncertainty estimated from VO can benefit the depth completion process. Since the VOID dataset does not provide uncertainty estimation for each input point, we adopt the uniform uncertainty estimation method from (Zhang and Ye, 2020) to compute the initial uncertainty for the input sparse depth. This estimated uncertainty is then fed into the first NCNN layer to train a baseline model. We report the results in Table 3 (denoted as *w/VO conf. init.*). After model convergence, the accuracy of the estimated depth significantly drops, with a large iRMSE indicating a high number of outliers. These observations suggest that directly incorporating the uncertainty from a model-based VO does not align well with the NCNN. We believe that employing a deep VO framework and training in an end-to-end manner may yield better results.

### 5.3 Evaluation of mapping performance

We adopt RTAB-Map (Labbé and Michaud, 2019) as the mapping module and utilize a voxel grid resolution of 0.01 m to store map for each sequence. We use either the ground truth pose (in VOID) or the pose from the V-SLAM algorithm (in our own sequence) in the mapping module to fairly assess the impact of depth estimation from different methods on mapping performance. The ground truth is generated using the ground truth depth with offline post-processing. Following (Stathoulopoulos et al., 2023), we use CloudCompare, an open-source point cloud processing software, to first align each map and then calculate the distance between the two point clouds (Mean Dist. (m) ) and the standard deviation (Var.) as error metrics for mapping.

#### 5.3.1 VOID

We evaluated the mapping performance of IU-DC and VI-Depth on three distinct sequences from the VOID dataset under two levels of input sparsity: ∼0.15% and ∼0.05% . The results are summarized in Table 4. Under normal sparsity (∼0.15%) , IU-DC outperforms VI-Depth by almost twice the error metrics across all sequences, with accuracy improvements ranging from 27% to 52% , and nearly half the variance. Moreover, when the input sparsity is further reduced to ∼0.05% —representing extreme scenarios where the robot may operate in low-texture regions—IU-DC continues to significantly outperform VI-Depth. These findings indicate that, despite IU-DC being only 2.8% the size of VI-Depth, it is better suited for robotic mapping tasks, as it facilitates the generation of more precise spatial maps through its uncertainty-aware approach.

**TABLE 4:** Mapping accuracy in VOID sequences.

Metric	*desktop*		*office*		*visionlab*	
	IU-DC	VI-Depth	IU-DC	VI-Depth	IU-DC	VI-Depth
∼ *0.15%*						
Mean Dist.	**0.08**	0.11	**0.09**	0.19	**0.11**	0.22
Var.	**0.03**	0.05	**0.06**	0.14	**0.06**	0.17
∼ *0.05%*						
Mean Dist.	**0.09**	0.20	**0.10**	0.23	0.18	0.23
Var.	**0.04**	0.17	**0.09**	0.21	**0.15**	**0.17**

^a^ ∼0.15% and ∼0.05% indicate input sparsity. Bold numbers indicate the best performance.

#### 5.3.2 Long trajectory sequence in the study office

Most sequences in the VOID dataset are recorded in constrained areas with short trajectories. To evaluate our method in a more open environment with longer trajectories, which are more common scenarios encountered by mobile robots, we conducted an experiment in a large student office using a handheld Intel RealSense D435i depth camera. We obtain the pose using the open-source V-SLAM algorithm provided by RealSense and use depth images from the D435i as ground truth (same as in the VOID dataset) for both network training and inference. To generate the input for the network, we follow the method described in (Wofk et al., 2023) running the VINS-Mono feature tracker front-end (Qin et al., 2018) to obtain sparse feature locations and then sampling ground truth depth at those locations. Both the camera image and depth image have a resolution of 480×640 , with approximately 500 points used in the input depth image.

**FIGURE 9:**
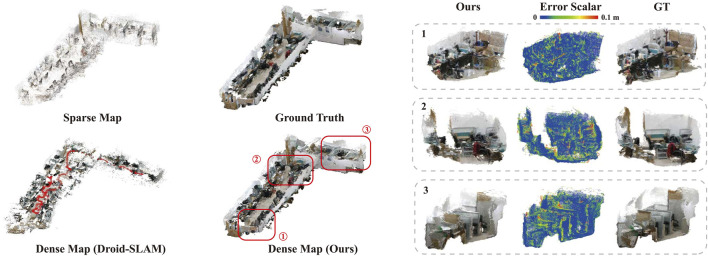
Evaluation of the Mapping Performance. The left part presents the maps generated by sparse VO, Droid-SLAM, and our method, while the right part shows three zoomed-in sections of our map with the associated error distribution. The Dense Map (ours) covers 78% volumes of the Ground Truth, whereas the Sparse Map covers only 1.5% .

The visualization of the map generated by sparse VO, Droid-SLAM (dense VO) (Teed and Deng, 2021), our method, and the ground truth is presented in Figure 9. Our method achieves significant completeness with 50× reconstruction volumes compared to the Sparse Map and captures detailed structural information of the environment with high accuracy. Although Droid-SLAM shows improvement in completeness over sparse VO, there is still considerable missing spatial information. To further validate the impact of uncertainty filtering approach, we conduct a comparative study using the raw depth output from IU-DC in the mapping module. We consider the distance between maps and the ground truth within 0.05 m as correct volumes, while the rest are classified as false volumes. From the results in Table 5, the map constructed using filtered depth shows higher accuracy and greater consistency with the ground truth. Although using raw depth increases reconstruction volumes, it has a 28% error rate, whereas filtered depth only has 10% . A video demonstrating the real-time mapping performance using our proposed approach can be found in the Supplementary Materials.

**TABLE 5:** Mapping accuracy in study office.

Input	Correct vol	False vol	Mean dist	Var
*raw_depth*	9.55	3.67	0.11	0.09
*filtered_depth*	7.05	0.87	0.07	0.04

^a^ Vol. Is in $m^{2}$ .

#### 5.3.3 Application in map alignment

**FIGURE 10:**
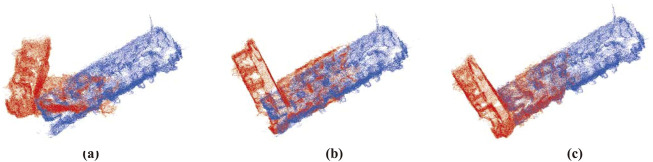
Alignment of maps from two robot coordinates. **(a)** Initial relative pose. **(b)** Alignment with maps generated by VO. **(c)** Alignment with maps generated by our method. We use the ground truth map with a voxel resolution downsampled to 0.05 m for visualization.

We conducted two trajectories, each mapping half of the office with a small overlapping area, to simulate the case of a two-robot system. First, we align the two maps using the ground truth pose and then introduce random translations and rotations to simulate potential false relative poses in real-world scenarios, which is presented in Figure 10A. We align the two local maps using the transformation matrix calculated by GICP (Segal et al., 2009), utilizing the same overlapping region of the sparse map generated by VO, as shown in Figure 10B, and the dense map generated by our method, as shown in Figure 10C. The results show that directly using the sparse map results in false alignment due to the map being too sparse, with missing reliable features. In contrast, the completed map recovers most of the environmental structures, providing sufficient features for accurately aligning the two maps. Though the experiment is conducted with two robots, it can be easily extended to a multi- or even swarm-robot system.

## 6 Conclusion

In this work, we propose a novel IU-DC to complete the extremely sparse depth data from VO, enhancing spatial perception through dense mapping of the environment. We extend NCNN into an image-guided approach with a specifically designed image feature integration mechanism and an ISM-based uncertainty estimation method to encode both color and spatial features, demonstrating superior performance in both depth and uncertainty estimation. The uncertainty-aware depth output from IU-DC exhibits outstanding performance compared to other VO depth completion methods in the context of robot mapping, achieving 50× more reconstructed space than the original sparse map and 78% coverage of the ground truth with high accuracy. IU-DC is also computationally efficient and requires limited memory consumption, showcasing its potential deployment on mobile robots.

The major limitation of our work, similar to other VO depth completion methods, is the reliance on VO to generate an accurate initial depth estimation. A promising future direction would be to generate uncertainty estimations for both input and output depth, and further apply optimization techniques to tightly couple these uncertainties with the depth estimation from the network.

## Data Availability

The original contributions presented in the study are included in the article/Supplementary Material, further inquiries can be directed to the corresponding authors.
